# Critically low accuracy of MRI in knee injury diagnosis in Afghanistan healthcare system: a comparative study with arthroscopy and physical examination

**DOI:** 10.1186/s12891-026-09696-y

**Published:** 2026-03-07

**Authors:** Ahmad Shakib Mohebi, Meena Obaide, Nommanudien Naibkhil, Zainuddin Mansoor, Abdul Waleed Yousufzai, Emal Wardak

**Affiliations:** 1 Department of Orthopedics Surgery and Traumatology, Ariana Medical Complex, Kabul, Afghanistan; 2 Department of orthopedic surgery and traumatology, Wazir Mohammad Akbar Khan Hospital, Kabul, Afghanistan; 3 Department of orthopedic surgery and traumatology, Kabul Atlas Hospital, Kabul, Afghanistan; 4 khatam Al-Nabieen Medical University, Kabul, Afghanistan; 5 Department of Biochemistry, Faculty of Pharmacy, Kabul University, Kabul, Afghanistan; 6 Department of Research, Ariana Medical Complex, Kabul, Afghanistan; 7 Department of Orthopedics Surgery and Traumatology, Afghan National Police Hospital, Kabul, Afghanistan

**Keywords:** Knee trauma, MRI accuracy, Arthroscopy, Physical examination, Afghanistan

## Abstract

**Background:**

Knee injuries, particularly those involving the meniscus and anterior cruciate ligament (ACL), are common musculoskeletal disorders that can lead to significant clinical and functional impairment. Accurate diagnosis is crucial for effective management and recovery. This study aimed to compare the diagnostic efficacy of physical examination (PE) and magnetic resonance imaging (MRI) for detecting meniscus tears and ACL injuries, at Afghanistan using arthroscopy as the reference standard.

**Methodology:**

A retrospective cross-sectional study was conducted on 377 patients with meniscus tears and 570 patients with ACL injuries at multiple hospitals in Kabul, Afghanistan. Diagnostic performance metrics, including sensitivity, specificity, accuracy, and predictive values, were calculated for PE and MRI.

**Results:**

The results revealed that PE demonstrated superior diagnostic performance compared to MRI. For meniscus tears, PE exhibited a sensitivity of 91.0%, specificity of 91.0%, and overall accuracy of 91.0%, with a positive predictive value (PPV) of 91.92% and a negative predictive value (NPV) of 90.0%. The diagnostic odds ratio (DOR) for PE was 102.12, indicating high reliability. In contrast, MRI showed significantly lower diagnostic accuracy for meniscus tears, with a sensitivity of 42.0%, specificity of 14.1%, and overall accuracy of 28.91%. The DOR for MRI was only 0.1188, reflecting poor diagnostic utility. Similarly, for ACL injuries, PE outperformed MRI with a sensitivity of 87.4%, specificity of 82.2%, and overall accuracy of 86.1%. The PPV and NPV for PE were 93.7% and 68.2%, respectively, with a DOR of 32.1. MRI, on the other hand, showed a sensitivity of 76.9% and a specificity of 8.5%, resulting in an overall accuracy of 60.0% and a DOR of 0.310. Receiver operating characteristic (ROC) curve analysis further confirmed the diagnostic superiority of PE, with an area under the curve (AUC) of 0.910 for meniscus tears and 0.848 for ACL injuries, compared to MRI’s AUC of 0.281 and 0.427, respectively. Combining PE and MRI using logical OR and AND analyses improved sensitivity but reduced specificity, indicating that PE alone remains the more reliable diagnostic tool.

**Conclusion:**

This study highlights the diagnostic superiority of PE over MRI for meniscus tears and ACL injuries, particularly in resource limited sittings specifically at Afghanistan PE demonstrated higher diagnostic accuracy than MRI for both ACL and meniscus injuries, highlighting its reliability as a cost-effective first-line tool in resource-limited settings like Afghanistan.

**Supplementary Information:**

The online version contains supplementary material available at 10.1186/s12891-026-09696-y.

## Introduction

Knee injuries, particularly those affecting the meniscus and anterior cruciate ligament (ACL), are among the most common musculoskeletal conditions, often leading to pain, instability, and long-term functional limitations if not accurately diagnosed. Early and precise diagnosis is therefore critical to guide treatment and improve outcomes. Clinicians typically rely on a combination of physical examination (PE), magnetic resonance imaging (MRI), and arthroscopy to evaluate these injuries. While arthroscopy is the gold standard, its invasive nature limits widespread use, especially in routine or resource-constrained settings.

A growing body of literature has compared the diagnostic performance of PE and MRI across different contexts. In high-resource settings, a meta-analysis reported pooled MRI sensitivity and specificity for ACL tears of 87% and 93%, respectively, with similar high accuracy for medial meniscus tears(89% sensitivity, 88% specificity) and somewhat lower values for lateral meniscus tears 78% sensitivity, 95% specificity [1]. In a prospective study of 72 patients, PE demonstrated strong performance, particularly for ACL injuries 88.7% sensitivity, 94.7% specificity, while MRI showed higher sensitivity but variable specificity for meniscal lesions e.g., medial meniscus 92.5% sensitivity, 62.5% specificity [2]. Larger series confirm these findings: an analysis of 543 patients found MRI accuracy of 85.8% for medial meniscus tears and 83.1% for lateral meniscus tears [3]. However, studies from resource-limited regions show more inconsistent results. For example, a Pakistani cohort reported MRI sensitivity for ACL injuries of 93.4% but much lower accuracy for lateral meniscus tears 37.5% sensitivity, 91.5% specificity [4]. These differences suggest that diagnostic performance may vary widely depending on imaging technology, radiologist training, and healthcare infrastructure.

Despite these international data, there is a knowledge gap in Afghanistan. MRI is widely used but often performed with outdated machines and interpreted by radiologists without musculoskeletal specialization, raising concern that diagnostic accuracy may be much lower than reported in high-resource studies. To date, no published research from Afghanistan has systematically compared PE and MRI against arthroscopy. Addressing this gap is essential, as reliance on low-accuracy MRI could lead to misdiagnosis and inappropriate management.

This study therefore aimed to evaluate and directly compare the diagnostic efficacy of PE and MRI for meniscal and ACL injuries, using arthroscopy as the gold standard, in a resource-limited Afghan setting.

## Methodology

This retrospective cross-sectional study was conducted at three orthopedic centers in Kabul, Afghanistan: Ariana Medical Complex, Kabul Atlas Hospital, and Wazir Mohammad Akbar Khan General Hospital, between January 2022 to December 2024.

Patients were included if they presented with clinical suspicion of ACL or meniscus injury, defined as knee pain, swelling, instability, or mechanical symptoms (locking, catching, or giving way) following acute trauma, together with at least one abnormal clinical examination finding.

Inclusion criteria: Age 15–55 years, clinical suspicion of ACL or meniscus injury, underwent both PE and MRI prior to arthroscopy, and availability of complete medical records.

Exclusion criteria: Previous knee surgery, associated fractures or multi-ligamentous knee injuries, systemic conditions affecting joint integrity (e.g., rheumatoid arthritis), and Incomplete records.

### Physical examination

Physical examination was performed by two senior orthopedic surgeons (> 5 years’ experience in knee surgery). Each patient was examined clinically prior to MRI and arthroscopy.

*ACL tests performed*: Lachman test, Anterior Drawer test, Pivot Shift test, and Lever Sign test.

*Meniscus tests performed*: McMurray’s test, Apley’s Compression test, Joint Line Tenderness, and Thessaly test.

### Criteria for positive PE

- ACL tear: At least two positive ACL-specific tests were required.
- Meniscus tear: At least two positive meniscus-specific tests were required.

Surgeons were aware of the patient’s clinical history but did not review MRI reports before conducting PE. Inter- and intra-observer reliability was not formally measured, which is acknowledged as a study limitation.

MRI scans were performed at various radiology centers in Kabul. Due to variability across facilities, exact scanner manufacturer, field strength, and sequence details were not consistently available Most scanners in use are low-field systems (≤ 0.4 Tesla), with only one 1.5 Tesla machine currently available at the French Medical Institute for Mothers and Children (FMIC). Crucially, the majority of MRI reports were interpreted remotely by radiologists in Pakistan and India and returned electronically to Afghan hospitals. For the purposes of this study, diagnostic interpretation was based on the final written radiology reports contained in patient files, rather than re-reading the images. This reflects the real-world diagnostic process in Afghanistan, but also represents a limitation, as differences in scanner quality and radiologist expertise may have contributed to the poor diagnostic accuracy observed.

Arthroscopy was performed by orthopedic surgeons with more than 5 years’ arthroscopic experience. Surgeons were aware of MRI reports and patient’s clinical history and PE findings. Arthroscopy was both diagnostic and therapeutic, serving as the reference standard. Findings were documented in detail, including whether the ACL tear was partial or complete and the type of meniscal tear (longitudinal, radial, horizontal, root, ramp).

 Data were collected retrospectively from hospital records and included: Demographics: age, sex, injured side, Clinical findings, mechanism of injury, detailed results of each PE test, MRI data report outcome (positive/negative for ACL and/or meniscus tear), and unavailable technical information, Arthroscopy findings: type and extent of ACL tear, meniscus tear morphology and location, Treatment details: whether arthroscopy was purely diagnostic or combined with therapeutic repair.

### Statistical analysis

Diagnostic accuracy measures (sensitivity, specificity, overall accuracy, PPV, NPV, likelihood ratios, diagnostic odds ratio) were calculated using arthroscopy as the reference standard. Agreement between PE and MRI was measured with Cohen’s kappa coefficient. McNemar’s test was applied to compare paired proportions of diagnostic accuracy, and receiver operating characteristic (ROC) curve analysis was performed to evaluate overall diagnostic performance.

## Results

A total of 947 patients with suspected knee injuries were initially reviewed. Of these, 331 cases were excluded due to incomplete records or lack of definitive arthroscopic confirmation. The final study population therefore consisted of 616 patients, including 570 with ACL injuries and 313 with meniscal injuries (with some patients having combined injuries).

For each patient, MRI reports, clinical examination findings, and arthroscopic operative findings were collected separately. All data were documented on structured collection sheets and subsequently entered into Microsoft Excel for statistical analysis.

The majority of participants were male (84.1% of meniscus cases, 82.6% of ACL cases). The most affected age group was 21–30 years (54.9% for meniscus, 56.5% for ACL). Right-sided injuries were slightly more common (51.1% and 51.8%, respectively).

Injuries were primarily due to sports and trauma. Specifically, 62.4% of meniscus and 65.8% of ACL injuries were sports-related (e.g., football, wrestling), while 29.1% of meniscus and 27.0% of ACL injuries were caused by road traffic accidents or falls. A smaller proportion (8.5% meniscus, 7.2% ACL) occurred due to other causes (e.g., workplace accidents).

### Diagnostic accuracy for meniscal tears

The PE for detecting meniscal tears demonstrated robust performance. It exhibited a sensitivity and specificity of 91.0%, indicating its effectiveness in accurately identifying both positive and negative cases. The overall accuracy of PE was also 91.0%, supported by a positive predictive value (PPV) of 91.92% and a negative predictive value (NPV) of 90.0%. The high F1 score of 91.6% reflects a strong balance between precision and recall, while the diagnostic odds ratio (DOR) of 102.12 underscores the reliability of PE as a diagnostic tool (Tables 1 and 2).

**Table 1:** Characteristics of the study population

Variable	Meniscus Tear		ACL injuries	
	N	%	N	%
Gender				
Female	60	15.9	99	17.4
Male	317	84.1	471	82.6
Side of injury				
Right	204	51.1	295	51.8
Left	173	45.9	275	48.2
Age				
20 and below	82	21.8	113	19.8
21-30	207	54.9	322	56.5
31-40	64	17	96	16.6
41-50	15	4	24	4.2
Above 50	9	2.4	15	2.6

In contrast, MRI showed significantly lower diagnostic accuracy for meniscal tears. Its sensitivity was 42.0%, and its specificity was only 14.1%, resulting in an overall accuracy of 28.91%. The PPV and NPV for MRI were 35.59% and 17.73%, respectively, with an F1 score of 38.7%. The DOR for MRI was a mere 0.1188, indicating poor diagnostic utility. The McNemar test (*p* = 0.023) revealed a statistically significant difference in performance between PE and MRI, favoring PE. Additionally, Cohen’s kappa coefficient for agreement between PE and MRI was − 0.429 (*p* < 0.001), indicating that the two methods had more disagreement than would be expected by random chance (Table 2).

**Table 2:** Performance of Physical Examination and MRI in Detecting Meniscus Tear and Anterior Cruciate Ligament Injury, Using Arthroscopy as the Gold Standard

	Test	Sensitivity	Specificity	Accuracy	PPV	NPV	F1 Score	MCC	LR+	LR-	DOR
1	Physical Examination Performance for Meniscus Tear against gold standard arthroscopy	91.0%	91.0%	91.0%	91.92%	90.0%	91.6%	0.794	10.11	0.099	102.12
2	MRI Performance for Meniscus Tear against gold standard Arthroscopy	42.0%	14.1%	28.91%	35.59%	17.73%	38.7%	-0.374	0.488941	4.113475	0.1188
3	Physical Examination Performance for ACL injury against gold standard arthroscopy	87.4%	82.2%	86.1%	93.7%	68.2%	90.4%	0.663	4.91	0.153	32.1
4	MRI Performance for ACL injury against gold standard Arthroscopy	76.9%	8.5%	60.0%	71.9%	10.8%	74.3%	-0.163	0.840	2.71	0.310

*PPV* Positive Predictive Value, *NPV* Negative Predictive Value, *MCC* Matthews Correlation Coefficient, *LR+* Positive Likelihood Ratio, *LR-* Negative Likelihood Ratio, *DOR* Diagnostic Odds Ratio

### Diagnostic accuracy for ACL injuries

In the assessment of ACL injuries, PE demonstrated superior performance compared to MRI. The sensitivity and specificity of PE were 87.4% and 82.3%, respectively, resulting in an overall accuracy of 86.1%. The positive PPV was 93.7%, while the NPV was 68.2%. With an F1 score of 90.4%, PE showcased a strong balance between precision and recall. The DOR of 32.1 further confirmed the reliability of PE (Table 2).

In contrast, MRI exhibited a moderate sensitivity of 76.9% but an extremely low specificity of 8.5%, leading to an overall accuracy of just 60.0%. The PPV for MRI was 71.9%, and the NPV was significantly lower at 10.8%, with an F1 score of 74.3%. The DOR was only 0.310, indicating poor diagnostic performance. The McNemar test (*p* < 0.001) revealed a statistically significant difference between PE and MRI, with PE clearly demonstrating superior diagnostic accuracy (Table 2). Furthermore, Cohen’s kappa coefficient for agreement between the two methods was − 0.057 (*p* = 0.158), reflecting very poor concordance.

### ROC analysis

Receiver operating characteristic (ROC) curve analysis further illustrated the enhanced diagnostic accuracy of PE for knee injuries. For meniscal tears, PE achieved an area under the curve (AUC) of 0.910, indicating excellent performance, while MRI had a significantly lower AUC of 0.281, suggesting very poor diagnostic capability. Similarly, PE recorded a high AUC of 0.848 for ACL injuries compared to MRI’s low AUC of 0.427. These findings underscore the diagnostic superiority of PE over MRI for knee injuries.

### Combined diagnostic approaches

Combining PE and MRI using logical OR increased meniscal tears sensitivity, rising to 94.5%, though the specificity dropped to 9.0%. For ACL injuries, sensitivity improved to 76.9%, but specificity fell to 8.5%. Conversely, using logical AND enhanced specificity at the expense of sensitivity. For meniscal tears, the sensitivity and specificity were 38.5% and 96.0%, respectively, while for ACL injuries, they were 69.4% and 87.2% (Table 3). These findings indicate that although combining the tests can improve certain diagnostic metrics, PE alone remains the more reliable tool for diagnosis.

**Table 3:** Cross-correlation of physical examination and MRI with arthroscopy

Logical OR					
	Sensitivity (%)	Specificity (%)	Accuracy (%)	PPV (%)	NPV (%)
Meniscus tears	94.5	9.0	54.4	54.0	59.3
ACL injuries	76.9	8.5	60.0	71.9	10.8
Logical AND					
Meniscus tears	38.5	96.0	65.5	91.6	58.0
ACL injuries	69.4	87.2	73.9	94.4	48.4

OR Positive if either PE or MRI positive, AND Positive only if both PE and MRI positive, PPV Positive Predictive Value, NPV Negative Predictive Value, ACL anterior cruciate ligament

## Discussion

The accurate diagnosis of knee injuries, particularly those involving the ACL and meniscus, is critical for effective treatment and prevention of long-term complications such as osteoarthritis. In resource-limited settings like Afghanistan, where access to advanced diagnostic tools is often constrained, identifying reliable and cost-effective diagnostic methods is paramount. This study provides valuable insights into the comparative diagnostic efficacy of PE and MRI for ACL and meniscal injuries, using arthroscopy as the reference standard.

PE demonstrated remarkable diagnostic accuracy in this study, with sensitivity and specificity exceeding 90% for meniscal tears and 87% for ACL injuries. These findings reinforce the value of PE as a reliable, cost-effective, and accessible diagnostic tool, particularly in resource-limited settings. PE allows clinicians to assess joint stability, range of motion, and specific clinical signs such as joint line tenderness or ligamentous laxity, which are critical for accurate diagnosis [5]. However, PE is not without limitations. In athletes with well-developed musculature, PE may yield false-negative results due to the stabilizing effect of strong muscles around the knee joint. For example, an anterior drawer test may fail to detect an ACL tear in such individuals, as the surrounding muscles can mask joint instability. Conversely, false-positive results may occur due to confounding factors such as pain or swelling, which can mimic the signs of an ACL or meniscal injury [6]. These limitations highlight the importance of combining PE with advanced diagnostic tools like MRI or arthroscopy in complex cases.

The high diagnostic values of PE in our study should be interpreted with caution, as the retrospective design and absence of inter-observer reliability testing may have contributed to inflated performance. However, several contextual factors unique to our patient population likely explain the elevated PE accuracy. Most patients presented late often between six months and two years after injury by which time quadriceps weakness, atrophy, and functional instability were pronounced, making clinical signs easier to detect. In addition, delayed arthroscopic confirmation increased diagnostic certainty at the time of surgery, though this also magnified discrepancies with MRI reports that were frequently inconsistent or mismanaged. Importantly, our findings are not intended to position PE as universally superior to MRI, but rather to emphasize the contextual limitations of MRI in Afghanistan’s healthcare setting.

Recent studies report MRI sensitivity and specificity exceeding 80–90% for both ACL and meniscal tears, highlighting our results as outliers [7]. This discrepancy likely reflects several contextual factors: most MRI machines in Afghanistan operate at ≤ 0.4 Tesla with non-standardized protocols, radiology reports are often prepared by clinicians with variable musculoskeletal expertise, and some scans were outsourced to neighboring countries, introducing reporting inconsistencies. Additionally, certain tear types, such as root and ramp lesions, are notoriously difficult to detect even in high-resource settings [8]. In our study, MRI demonstrated substantially lower sensitivity (76.9% for ACL injuries, 42.0% for meniscal tears) and specificity (8.5% for ACL injuries, 14.1% for meniscal tears) compared to physical examination. These findings align with previous reports highlighting the limitations of MRI, which include dependency on machine quality, the type and location of the tear, and the expertise of the interpreting radiologist [9] specially in developing countries. In contrast, several international studies have reported superior diagnostic performance of physical examination over MRI, with specificity for ACL tears ranging between 83.3% and 98.6% and sensitivity between 88.4% and 100%, and for meniscal tears, specificity between 71.4% and 95% and sensitivity between 84.2% and 85.7% [10]. Overall, these results underscore the limited reliability of MRI for musculoskeletal injuries in the Afghan healthcare setting. Arthroscopy remains the gold standard for diagnosing ACL and meniscal injuries, offering both diagnostic and therapeutic benefits. It provides direct visualization of intra-articular structures, allowing for accurate assessment of injury severity and concurrent pathologies [11]. Arthroscopy is particularly useful in cases where non-invasive diagnostic methods, such as PE and MRI, yield inconclusive results or when surgical intervention is required [12]. However, its invasive nature and associated risks, such as infection and anesthesia complications, limit its use to more complex cases. In this study, arthroscopy confirmed the diagnostic superiority of PE over MRI, highlighting the importance of clinical examination as a cost-effective and reliable diagnostic tool, particularly in settings where advanced imaging is unavailable.

The low diagnostic accuracy of MRI in our study can be attributed to several factors, including the lack of updated MRI machines and the limited expertise of radiologists in Afghanistan. Most MRI scans in the country are interpreted by radiologists with insufficient training in musculoskeletal imaging, particularly for complex knee injuries. Additionally, many MRI reports are outsourced to neighboring countries like India and Pakistan, where advanced imaging services are more readily available. This reliance on external sources for diagnostic reports underscores a critical gap in the capacity of South Asian radiologists, who often lack the necessary training and expertise to interpret complex imaging results effectively [13].

In this study, combining PE and MRI using logical OR analysis increased sensitivity, while logical AND analysis improved specificity, reflecting the classical trade-off between these approaches. Similar findings have been described in other studies, where the combination of PE and MRI enhanced diagnostic accuracy compared with either modality alone [14]. Kotian et al. [15] reported that thorough clinical assessment combined with MRI provided diagnostic performance comparable to MRI alone while emphasizing the value of experienced clinical examination; El-Hagrasy et al. [9] demonstrated high MRI accuracy for meniscal pathology but noted that clinical correlation is essential in discordant cases; and Van Dyck et al. [8] analyzed discordant findings between 3T MRI and arthroscopy and highlighted unavoidable and equivocal errors that can lead to false negatives or false positives. These findings suggest that PE offers immediate bedside diagnostic value while MRI contributes complementary structural detail to refine diagnosis and guide surgical planning. In resource-limited settings such as Afghanistan, optimizing this combination requires practical solutions: strengthening radiology training, standardizing MRI protocols (preferably ≥ 1.5 Tesla), and developing collaborative pathways between radiologists and Orthopaedic surgeons. With such improvements, PE and MRI can act synergistically to enhance diagnostic reliability even in low-resource contexts.

To address these challenges, there is an urgent need to invest in the training and education of Afghan radiologists, particularly in the interpretation of knee MRI. Research by Alkhayat and Ashkanani [16] has demonstrated that targeted training programs for radiologists can significantly improve diagnostic accuracy and reduce reliance on foreign reports. Establishing comprehensive training programs, along with continuous professional development, would allow Afghan radiologists to build expertise and provide more accurate, localized diagnoses. Strengthening the capacity of local healthcare systems to perform and interpret imaging studies—supported by the gradual introduction of updated MRI machines—could enhance the overall quality of healthcare, reduce delays in diagnosis, and contribute to better patient outcomes.

## Conclusion

Physical examination demonstrated higher diagnostic accuracy than MRI for ACL and meniscus injuries in Afghanistan. While MRI remains valuable, its utility is limited by outdated equipment and insufficient radiologist training. PE is therefore a reliable, accessible, and cost-effective first-line tool. Investments in MRI infrastructure and radiologist education are urgently needed to improve diagnostic accuracy in Afghanistan. 

## Supplementary Information

Supplementary Material 1.

## Data Availability

The data that support the findings of this study are available from the corresponding author upon reasonable request.

## Abbreviation

ACL: Anterior Cruciate Ligament
PE: Physical Examination
MRI: Magentic Resonance Imaging
PPV: Positive Predictive Value
NPV: Negative Predictive Value
ROC: Receiver Operating Characteristic
AUC: Area Under the Curve
Logical OR: Positive if either PE or MRI positive
AND: Positive only if both PE and MRI positive
DOR: Diagnostic Odds Ratio
